# Large DNA fragment IS*Ec9*-mediated transposition during natural transformation allows interspecies dissemination of antimicrobial resistance genes

**DOI:** 10.1007/s10096-025-05113-9

**Published:** 2025-03-28

**Authors:** Sara Domingues, Tiago Lima, Corentin Escobar, Julie Plantade, Xavier Charpentier, Gabriela Jorge da Silva

**Affiliations:** 1 Faculty of Pharmacy, University of Coimbra, Coimbra, Portugal; 2 CNC-UC - Center for Neuroscience and Cell Biology, University of Coimbra, Coimbra, Portugal; 3 CIVG -Vasco da Gama Research Center, EUVG – Vasco da Gama University School, Coimbra, Portugal; 4 CIRI, Centre International de Recherche en Infectiologie, Inserm, U1111, Université Claude Bernard Lyon 1, CNRS, UMR5308, École Normale Supérieure de Lyon, Univ Lyon, Villeurbanne, 69100 France

**Keywords:** Antimicrobial resistance, CTX-M, IS*Ec9*, Natural transformation, Transposition

## Abstract

**Purpose:**

Antimicrobial resistance poses a significant global health challenge, contributing to a lack of effective therapeutic agents, especially against Gram-negative bacteria. Resistance dissemination is accelerated by horizontal gene transfer (HGT) mechanisms. The extended-spectrum beta lactamases CTX-M confer resistance to several beta-lactams, are usually embedded into plasmids and thought to be mainly disseminated by conjugation. However, an increasing number of isolates carry these enzyme-encoding genes in the chromosome, suggesting that they can spread by other means of HGT. In this study, we aimed to test the involvement of natural transformation in the chromosomal acquisition of a *bla*_CTX−M_ gene.

**Methods:**

Natural transformation assays were performed during motility on wet surfaces. Acquisition of foreign DNA by transformants was screened by antimicrobial susceptibility testing, polymerase-chain reaction (PCR) and whole genome sequencing (WGS).

**Results:**

*Acinetobacter baumannii* A118, a naturally competent clinical strain, was transformed with naked DNA from *Salmonella enterica* serovar Typhimurium Sal25, which was isolated from swine meat. The transformation occurred at low frequency (2.7 × 10^− 8^ ± 2.04 × 10^− 8^ transformants per recipient) and *bla*_CTX−M_ was acquired in one transformant, which was named ACI. WGS of the transformant revealed the acquisition of the *bla*_CTX−M−32_ as part of a ca. 36 Kb DNA fragment through an IS*Ec9*-mediated transposition event; various mobile genetic elements and other resistance genes were co-transferred. The *bla*_CTX−M−32_ gene was subsequently transferred within *A. baumannii* at a higher frequency (1.8 × 10^− 6^ ± 2.49 × 10^− 6^ transformants per recipient).

**Conclusion:**

Our results highlight the importance of natural transformation events in the dissemination of antimicrobial resistance genes and mobile genetic elements between and within species.

**Supplementary Information:**

The online version contains supplementary material available at 10.1007/s10096-025-05113-9.

## Introduction

Antimicrobial resistance represents one of the major threats to global public health, undermining the effectiveness of treatments and leading to higher mortality rates and healthcare costs associated with multidrug resistant infections [1]. The recently updated World Health Organization (WHO) list of pathogens that urgently need new antibiotics mainly includes Gram-negative bacteria, with *Acinetobacter baumannii* at the top of the critical group [2], due to the limited therapeutic options and the high rates of multidrug resistance. *A. baumannii* is also part of the ESKAPE pathogens [3]. Generally considered a nosocomial pathogen [4], *A. baumannii* can also be found in activated sludge, animals, food, sewage, soil and water [5–8], where it can cohabit with different bacterial species, including *Salmonella enterica* [9–11].

Amongst the resistance determinants, beta-lactamases are particularly concerning, especially in Gram-negative bacteria, as they confer resistance to the most widely used family of antibiotics, beta-lactams, including those considered as last resort therapeutic options. CTX-M enzymes are plasmid-encoded extended spectrum beta-lactamases (ESBL) commonly found worldwide [12], in isolates of human [13], animal [14] and environmental [15] origin. CTX-M are highly disseminated among *Enterobacterales* and dissemination by conjugation is well-established [16], but the prevalence in non-fermentative bacteria is less common [17]. CTX-M found in *A. baumannii* are usually chromosomally-encoded [17, 18] and the horizontal gene transfer (HGT) mechanisms involved in the acquisition are usually unknown; presence of these enzymes in *A. baumannii* reduces even more the therapeutic options against this multidrug-resistant pathogen. *A. baumannii* is able to undergo all HGT mechanisms [19–22], and this trait may be of major importance in the acquisition of its multidrug resistance profile. Although conjugation is considered a key driver of resistance genes spread among bacteria [23], natural transformation has been suggested as the main HGT mechanism involved in antibiotic resistance dissemination in *A. baumannii* [24]. Nonetheless few studies explore this intercellular mechanism [25], as well as the intracellular mobilization of the resistance genes remains scarcely known. Interspecies dissemination of *bla*_CTX−M−2_ from *Proteus mirabilis* to *A. baumannii* has been previously suggested [26], and the ability of *A. baumannii* to acquire *bla*_CTX-M-115_ from three *A. baumannii* clinical isolates by natural transformation followed by intracellular homologous recombination has been experimentally demonstrated [18]. Stable acquisition of a plasmid-encoding *bla*_CTX−M−2_ gene by *A. baumannii* A118 after DNA uptake by natural transformation has also been experimentally demonstrated [27].

The main aims of this study were to evaluate the involvement of natural transformation in interspecies *bla*_CTX−M_ genes dissemination, as well as to understand the intracellular mechanisms associated with their stable acquisition.

## Materials and methods

### Bacterial isolates

The naturally competent *A. baumannii* A118, a clinical antimicrobial susceptible isolate [28], *A. baylyi* BD413, a soil bacterium [29], *A. nosocomialis* 013 and *Acinetobacter* sp. 065, clinical multidrug resistant isolates [22], were used as recipient in natural transformation experiments. *S. enterica* serovar Typhimurium Sal25, isolated from swine meat and carrying a class 1 integron with the *aadA1* gene cassette, the *mcr-1* gene and a *bla*_CTX−M−1_ group gene in an IncHI2 plasmid [30, 31], was the source of the donor DNA. *A. baumannii* ACI, a transformant that acquired the *bla*_CTX−M−1_ group gene, was also used as donor DNA in subsequent transformation assays.

### DNA extraction

Genomic DNA for transformation assays was extracted from bacterial cultures using anion exchange columns (QIAGEN, Germany) according to the manufacturers protocol and resuspended in EB buffer, pH 8.5 (QIAGEN, Germany). The DNA concentration was measured with Nanodrop ND-1000 (Nanodrop Technologies, USA).

### Natural transformation assays

Natural transformation assays were performed during motility on wet surfaces, as previously described [32]. Briefly, a single colony of the recipient cell was suspended in 20 µl of sterile phosphate-buffered saline (PBS) and mixed with 20 µl of donor DNA (4 µg in water). This mixture was introduced into the semisolid Motility Medium (MM; 0.5% agar (Liofilchem), 5 g/l tryptone (Difco) and 2.5 g/l sodium chloride (Scharlau)) by stabbing the medium seven times with 2 µl of the transformation mixture each time. The plate was sealed with Parafilm to prevent dryness of the medium and incubated at 37°C for 24 h; bacteria were recovered from the medium surface and suspended in 1 ml of PBS, followed by selection on Luria-Bertani (LB; Fluka Analytica) agar with 30 µg/ml of cefotaxime (Sigma). Positive and negative controls were performed with *A. baumannii* homologous DNA [22] and water instead of donor DNA, respectively. Three independent transformation assays, each repeated in triplicate, were performed.

The transformation frequency (TF) was calculated as the ratio between the number of transformants and the number of viable recipient cells.

### Antimicrobial susceptibility testing

Antimicrobial susceptibility of recipient, donor and transformant cells was determined in Mueller-Hinton agar (Sigma-Aldrich) by disk diffusion with antibiotic disks (Oxoid) of amoxicillin (30 µg), amoxicillin-clavulanic acid (30 µg), aztreonam (30 µg), ceftazidime (30 µg), cefotaxime (30 µg), cefepime (30 µg), spectinomycin (10 µg), streptomycin (10 µg). The minimum inhibitory concentration (MIC) of cefotaxime and colistin was determined by broth microdilution in Mueller-Hinton and cation-adjusted Mueller-Hinton broth (CAMHB), respectively, according to EUCAST and CLSI guidelines [33, 34].

### Polymerase chain reaction (PCR)-based detection of antimicrobial resistance genes

DreamTaq Green PCR Master Mix (Thermo Fisher Scientific) was used in end-point PCRs; recipient and donor cells were used as negative and positive control, respectively. The acquisition of the *bla*_CTX−M_ gene by transformant ACI was screened with primers CTX-M/F’ + CTX-M/R’ [35]; co-acquisition of the *mcr-1* gene and the class 1 integron was checked with CLR5-F + CLR5-R [36] and 5’-CS + 3’-CS [37], respectively.

### Whole genome sequence

Whole genome sequencing (WGS) of *A. baumannii* A118, *S. enterica* Sal25 and transformant *A. baumannii* ACI were obtained with long reads sequencing technology (Oxford Nanopore Technologie, ONT). Genomic DNA was extracted with the DNeasy Blood and Tissue kit (Qiagen), following the manufacturer instructions. Libraries of DNA samples were prepared using the rapid barcoding kit 24 v14 (SQK-RBK114.24, ONT), following the manufacturer instructions. Libraries were sequenced using R10.4.1 flow cells on a MinION instrument. Data were acquired for 72 h, as advised by the manufacturer. Live basecalling was performed by the MinKNOW software using the superaccuracy model. A118 produced 76k reads totalling 183 Mb (46x coverage). ACI produced 92k reads for a total of 183 Mb and Sal25 generated 83k reads for a total of 239 Mb (47x coverage). A118 and ACI genomes were assembled with the ONT long reads using the Hybracter pipeline [38]. The Sal25 genome was obtained through the hybrid assembly of ONT reads and publicly available Illumina reads (Biosample: SAMEA3476857, European Nucleotide Archive) using Hybracter. Genomes were annotated with Bakta (version 1.8.2). Genomes were aligned and compared with the Mauve software (The Darling lab at the University of Technology Sydney) and Easyfig [39]. All genomes are available at NCBI under BioProject PRJNA1209693 with accession numbers CP178253 (A118), CP178251-CP178252 (Sal25) and CP178250 (ACI).

Sal25 plasmid was analysed with Phastest, to search for phage elements [40].

## Results

### Interspecies transfer and intracellular mobilization of antimicrobial determinants by natural transformation

We sought to test the possibility that *A. baumannii* could acquire antimicrobial resistant determinants by natural transformation, even from an unrelated species of different origin. The *A. baumannii* A118 strain was exposed to genomic DNA extracted from Sal25, a strain of the *Enterobacterales S. enterica* that was reported to encode resistance determinants to cephalosporins and colistin [30]. Following incubation and cefotaxime selection, A118 successfully acquired *S. enterica* Sal25 DNA at a frequency of 2.7 × 10^− 8^ ± 2.04 × 10^− 8^ transformants per recipient (Online Resource 1), a low frequency event, when compared with intraspecies transformation with genomic DNA from *A. baumannii* 121-1 [22] (TF = 1.4 × 10^− 5^ ± 1.98 × 10^− 5^ transformants per recipient). One tested transformant showed a susceptibility profile consistent with the acquisition of a CTX-M, namely reduced growth inhibition diameters to ceftazidime, cefotaxime, amoxicillin, aztreonam and cefepime (Online Resource 2), and the enlargement of the inhibition zone between cephalosporins and amoxicillin-clavulanic acid. The cefotaxime MIC of the transformant, which was named ACI, was > 512 mg/L, which correspond to a 128-fold increase, and the acquisition of the beta-lactamase was confirmed by PCR amplification of the gene. The susceptibility to the tested aminoglycosides (Online Resource 2) as well as the MIC to colistin did not change, which suggested that the transformant did not acquired the class 1 integron nor the *mcr-1* gene from the *Salmonella* donor, respectively [30, 31]; the non-acquisition of these elements was also confirmed by the absence of PCR amplification of the genes.

**Fig. 1:**
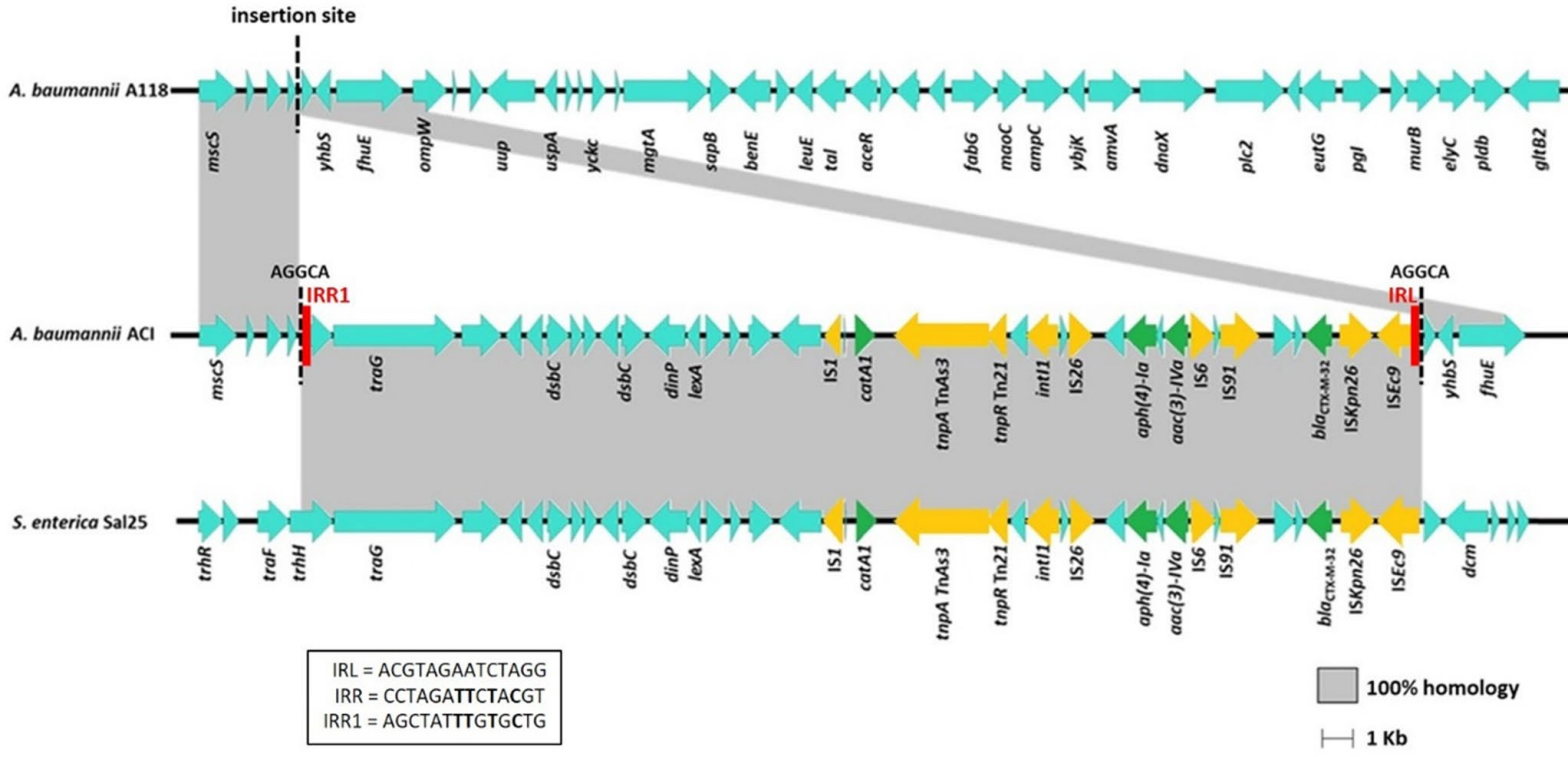
Schematic representation of the large DNA fragment acquired by IS*Ec9-*mediated transposition, which includes different resistance genes (coloured in green), and mobile genetic elements (coloured in yellow). Imperfect inverted repeats are coloured in red. The fragment was inserted into a non-coding region of the *A. baumannii* A118 (marked with dashed line), generating a 5 bp direct repeat (AGGCA). Homologous sequences with the donor and the recipient strains are highlighted in grey

WGS yielded the full genomes of the recipient, donor and transformant cells. A118 and ACI genomes consist of a single circular chromosome while Sal25 showed a chromosome (non-circularized) and a non-circularized 234 kb plasmid. WGS revealed that the *A. baumannii* ACI transformant acquired a 36,130 bp DNA fragment from the donor *S. enterica* Sal25, which was inserted into a non-coding region of the *A. baumannii* A118 chromosome; the only difference between ACI and A118 was the ca. 36 kb acquired fragment. Besides the ESBL-encoding gene, the fragment acquired by ACI included different resistance genes, namely the chloramphenicol O-acetyltransferase *catA1* gene and the aminoglycoside transferase genes *aph(4)-Ia* and *aac(3)-IVa*, as well as mobile genetic elements, including insertion sequences (IS) from several families and a class 1 integrase *intI* gene (Fig. 1). The complete sequence of the *bla*_CTX−M−1_ group gene identified the gene as the *bla*_CTX−M−32_ variant. IS*5*/IS*Kpn26* and IS*1380*/IS*Ec9* were detected upstream the *bla*_CTX−M−32_ gene. The acquired 36 Kb segment originates from a 230 Kb plasmid in Sal25, and with no homologous sequence to A118.

We detected a 5-bp (AGGCA) direct repeat (DR) flanking the acquired fragment, indicating the acquisition was caused by transposition of the DNA segment after uptake by natural transformation into the recipient cytoplasm. One of the 5-bp DR was located immediately adjacent to the left inverted repeat (IRL - ACGTAGAATCTAGG) of IS*Ec9* (also called IS*Ecp1*). The other DR was located immediately adjacent to the deduced right imperfect inverted repeat (IRR1 - AGCTATTTGTGCTG) of IS*Ec9*. The 14-bp IRR1 sequence detected in transformant *A. baumannii* ACI was part of the conjugal transfer protein-encoding gene *trhH*, has 4 base pairs identical to the perfect IRR and has a G residue at the 3’ end (Fig. 1).

### Intraspecies transfer of antimicrobial determinants by natural transformation

To test whether the acquired segment could spread by intragenus transfer, the *A. baumannii* ACI transformant was used in subsequent transformation assays as donor DNA. Among the four recipient isolates belonging to *A. baumannii*, *A. baylyi*, *A. nosocomialis* and *Acinetobacter* sp., only *A. baumannii* A118 was successfully transformed by *A. baumannii* ACI DNA, at a frequency of 1.8 × 10^− 6^ ± 2.49 × 10^− 6^ transformants per recipient (Online Resource 1). All tested transformants were resistant to ceftazidime, cefotaxime, amoxicillin and aztreonam (Online Resource 2), and were positive for the *bla*_CTX−M−32_ gene acquisition.

## Discussion

We have observed the transformation of *A. baumannii* A118 with naked DNA from *S. enterica* Sal25 at a frequency of 2.7 × 10^− 8^ ± 2.04 × 10^− 8^ transformants per recipient. Interspecies transformation of *A. baumannii* A118 with DNA from *Klebsiella pneumoniae* VA360 and Kb18 has been observed at similar frequencies [41]. *S. enterica* Sal25 was isolated from swine meat and *K. pneumoniae* VA360 and Kb18 are isolates of human clinical origin, demonstrating that naturally competent *A. baumannii* is able to acquire DNA from other species and sources.

*A. baumannii* ACI transformant acquired a long DNA fragment from the donor *S. enterica* Sal25, which contained several antimicrobial resistance genes and mobile genetic elements, especially insertion sequences. The *bla*_CTX−M−32_ gene was detected downstream of IS*5*/IS*Kpn26* and IS*1380*/IS*Ec9*, a genetic environment also previously detected in a *E. coli* isolated from a healthy bovine in Portugal [42], which may be common among isolates of animal origin.

Integration of the DNA taken up by natural transformation is usually based on homologous recombination. However, no homologous recombination events could be produced by uptake of this plasmid DNA from *S. enterica* Sal25, as there are no homologous sequences with A118. Neither complete nor partial phage elements were detected in the Sal25 plasmid, ruling out that the plasmid belongs to a phage-plasmid and can be packaged and delivered by a viral particle [43]. It is also unlikely that acquisition was due to a transduction event, as temperate phages have a narrow host range. The detection of a 5-bp DR flanking the 36 Kb DNA fragment and adjacent to the IS*Ec9* IRL and to an imperfect IRR suggests that the acquisition occurred by transposition mediated by IS*Ec9*. IS*Ecp1B*-mediated transposition with weakly related IRR, comprising 3 to 12 identical base pairs to the perfect IRR, has been observed and a G residue at the 3’ end of the IR was suggested as crucial for the transposition event [44], requirements fulfilled by the identified imperfect IRR. IS*Ec9* has been associated with the dissemination of *bla*_CTX−M_ genes and a single copy is able to promote one-ended transposition of downstream genes [45]. Based on WGS analysis, insertion of a *bla*_CTX−M−15_ into the chromosome of a clinical isolate of *K. pneumoniae* via an IS*Ec9* insertion sequence has been previously suggested [46]. In the same way, in an *Escherichia coli* isolated from blue mussels, the mobilizable trait of the chromosomal *bla*_CTX−M−14_ has been proposed due to the association with IS*Ec9* [47]. IS*Ec9* belongs to the IS*1380* family, a family that produces a 4–5 bp DR during transposition [48], which is in accordance with the target site duplication we have identified in our transformant. Most of the studies usually employ WGS to understand the mobility potential of the detected antibiotic resistance genes. In this study we experimentally demonstrated the intracellular acquisition of a *bla*_CTX−M−32_ by transposition as part of a large DNA fragment taken up by natural transformation. A previous study has also experimentally demonstrated acquisition of *bla*_CTX−M−2_ by *A. baumannii* A118 after uptake of plasmid DNA from *Proteus mirabilis* Prm9 by natural transformation; in that particular case the *bla*_CTX−M−2_-encoding plasmid was maintained as an extrachromosomal element [27]. Although typically involved in the mobilization of fragments no larger than 10 Kb [49–51], IS*Ec9-*mediated mobilization of a 14 Kb and a 41 Kb DNA fragments has been suggested [52]. In conclusion, the results show that *A. baumannii* could use natural transformation to acquire large DNA from unrelated species, even in the absence of homologous sequence. Instead, integration in the genome was caused by transposition occurring in the course of natural transformation, a low frequency event, likely involving the transient expression of the transposase and resolvase encoded by the donor DNA [53].

In comparison to the frequency of interspecies transformation between *A. baumannii* A118 and *S. enterica* Sal25, the intraspecies transfer of the ESBL-encoding gene exhibited a 20-fold increase, underscoring that HGT events that occur at low initial frequencies may subsequently be succeeded by additional occurrences at elevated frequencies, as previously documented in the dissemination of class 1 integrons [54]. The decreased interspecies recombination, as compared with intraspecies events, is called sexual isolation, and several factors may contribute to this barrier, including physical proximity of cells, DNA uptake machineries, and the existence of restriction-modification systems [55]. In fact, a recent study found that *A. baumannii* A118 has a restriction-modification system that recognizes the RGATCY motif, which limit the acquisition of unmethylated DNA or DNA with different methylation motifs [56]. The intraspecies acquisition of *bla*_CTX−M−32_ observed in this study likely occurs by homologous recombination, which is more frequent than transposition [54] and within isolates belonging to the same species [56, 57]. Intracellular acquisition of *bla*_CTX−M_ genes has also been previously suggested to occur by homologous recombination in *A. baumannii* [18].

The *bla*_CTX−M_ genes are usually plasmid-encoded, but there is a growing number of studies that report a chromosomal location in Gram-negative bacteria [18, 58–61]. In conclusion, the combined action of natural transformation and transposition, may explain this observation and lead to the stable incorporation of the acquired DNA into the recipient chromosome. Further studies are needed to clarify the impact of this mechanism in natural contexts, including biological and abiotic settings. Nonetheless, this work demonstrates that natural transformation allows intercellular spread of clinically important resistance genes even between such genetically-distant related bacteria like *A. baumannii* and *S. enterica*. The role of natural transformation as a pivotal mechanism facilitating the interspecies transmission of antimicrobial resistance is reinforced, highlighting the possible implications for the control of antimicrobial resistance dissemination within the One Health framework.

## Electronic supplementary material

Below is the link to the electronic supplementary material.

Supplementary Material 1

Supplementary Material 2

Supplementary Material 3

## Data Availability

Individual genomes are available on GenBank under accession numbers CP178250, CP178251, CP178252 and CP178253.
