# Supplementary material for: Large DNA fragment ISEc9-mediated transposition during natural transformation allows interspecies dissemination of antimicrobial resistance genes
Source: Eur J Clin Microbiol Infect Dis. 2025 Mar 28;44(6):1417–24. doi: 10.1007/s10096-025-05113-9 (PMC12116815; doi:10.1007/s10096-025-05113-9)
Supplement: Supplementary file 1 — Supplementary Material 1 [file 10096_2025_5113_MOESM1_ESM.docx]

**European Journal of Clinical Microbiology & Infectious Diseases**

**Title**: Large DNA fragment IS*Ec9*-mediated transposition during natural transformation allows interspecies dissemination of antimicrobial resistance genes

**Authors:** Sara Domingues^a,b^*, Tiago Lima^a,b,c^, Corentin Escobar^d^, Julie Plantade^d^, Xavier Charpentier^d^, Gabriela Jorge da Silva^a,b^

^a^ University of Coimbra, Faculty of Pharmacy, Coimbra, Portugal

^b^ CNC-UC - Center for Neuroscience and Cell Biology, University of Coimbra, and CiBB - Centre for Innovative Biomedicine and Biotechnology, University of Coimbra, Portugal

^c^ CIVG -Vasco da Gama Research Center, EUVG – Vasco da Gama University School, Coimbra, Portugal

^d^ CIRI, Centre International de Recherche en Infectiologie, Inserm, U1111, Université Claude Bernard Lyon 1, CNRS, UMR5308, École Normale Supérieure de Lyon, Univ Lyon, 69100, Villeurbanne, France

* Corresponding author; [saradomingues@ff.uc.pt](mailto:saradomingues@ff.uc.pt)

| **Donor DNA** | **Transformation assay** | **Transformation frequency (transformants/ recipient)** | | **Transformation frequency mean ± SD (transformants/ recipient)** |
| --- | --- | --- | --- | --- |
|  |  | Replicates | Mean |  |
| *Salmonella enterica* Sal25 | 1 | 1.61 x 10^-8^ | 2.00 x 10^-8^ | 2.7 x 10^-8^ ± 2.04 x 10^-8^ |
|  |  | 3.85 x 10^-9^ |  |  |
|  |  | 4.00 x 10^-8^ |  |  |
|  | 2 | 4.85 x 10^-8^ | 4.99 x 10^-8^ |  |
|  |  | 6.45 x 10^-8^ |  |  |
|  |  | 3.67 x 10^-8^ |  |  |
|  | 3 | 6.45 x 10^-9^ | 1.10 x 10^-8^ |  |
|  |  | 1.65 x 10^-8^ |  |  |
|  |  | 1.10 x 10^-8^ |  |  |
| *Acinetobacter baumannii* ACI | 1 | 4.60 x 10^-7^ | 3.39 x 10^-7^ | 1.8 x 10^-6^ ± 2.49 x 10^-6^ |
|  |  | 2.00 x 10^-7^ |  |  |
|  |  | 3.57 x 10^-7^ |  |  |
|  | 2 | 1.99 x 10^-7^ | 4.33 x 10^-7^ |  |
|  |  | 1.00 x 10^-7^ |  |  |
|  |  | 1.00 x 10^-6^ |  |  |
|  | 3 | 3.54 x 10^-6^ | 4.70 x 10^-6^ |  |
|  |  | 1.04 x 10^-5^ |  |  |
|  |  | 1.51 x 10^-7^ |  |  |
