# Supplementary material for: Large DNA fragment ISEc9-mediated transposition during natural transformation allows interspecies dissemination of antimicrobial resistance genes
Source: Eur J Clin Microbiol Infect Dis. 2025 Mar 28;44(6):1417–24. doi: 10.1007/s10096-025-05113-9 (PMC12116815; doi:10.1007/s10096-025-05113-9)
Supplement: Supplementary file 2 — Supplementary Material 2 [file 10096_2025_5113_MOESM2_ESM.docx]

**European Journal of Clinical Microbiology & Infectious Diseases**

**Title**: Large DNA fragment IS*Ec9*-mediated transposition during natural transformation allows interspecies dissemination of antimicrobial resistance genes

**Authors:** Sara Domingues^a,b^*, Tiago Lima^a,b,c^, Corentin Escobar^d^, Julie Plantade^d^, Xavier Charpentier^d^, Gabriela Jorge da Silva^a,b^

^a^ University of Coimbra, Faculty of Pharmacy, Coimbra, Portugal

^b^ CNC-UC - Center for Neuroscience and Cell Biology, University of Coimbra, and CiBB - Centre for Innovative Biomedicine and Biotechnology, University of Coimbra, Portugal

^c^ CIVG -Vasco da Gama Research Center, EUVG – Vasco da Gama University School, Coimbra, Portugal

^d^ CIRI, Centre International de Recherche en Infectiologie, Inserm, U1111, Université Claude Bernard Lyon 1, CNRS, UMR5308, École Normale Supérieure de Lyon, Univ Lyon, 69100, Villeurbanne, France

* Corresponding author; saradomingues@ff.uc.pt

| **Bacterial isolates** | **Inhibition zone diameter (mm)** | | | | | | | | **Minimum inhibitory concentration (mg/L)** | |
| --- | --- | --- | --- | --- | --- | --- | --- | --- | --- | --- |
|  | **AMC** | **AML** | **ATM** | **CAZ** | **CTX** | **FEP** | **S** | **SH** | **CTX** | **COL** |
| *Acinetobacter baumannii* A118 | 24 | 19 | 23 | 25 | 21 | 26 | 14 | 12 | 4 | 0.5 |
| *Salmonella enterica* Sal25 | 23 | 0 | 13 | 15 | 0 | 22 | 0 | 0 | >512 | 16 |
| Transformant *Acinetobacter baumannii* ACI* | 24 | 0 | 0 | 13 | 0 | 17 | 14 | 12 | >512 | 0.5 |
| Transformant *Acinetobacter baumannii* 1* | 24 | 19 | 23 | 23 | 8 | 25 | 14 | 12 | n.d. | n.d. |
| Transformant *Acinetobacter baumannii* 2* | 24 | 19 | 23 | 23 | 9 | 26 | 14 | 12 | n.d. | n.d. |
| Transformant *Acinetobacter baumannii* ACI-1* | 18 | 0 | 0 | 12 | 0 | 15 | n. d | n. d | >512 | n.d. |
| Transformant *Acinetobacter baumannii* ACI-2* | 18 | 0 | 0 | 13 | 0 | 17 | n. d | n. d. | >512 | n.d. |
| Transformant *Acinetobacter baumannii* ACI-3* | 22 | 0 | 0 | 13 | 0 | 17 | n. d. | n. d. | >512 | n.d. |
| AML – amoxicillin, ATM – aztreonam, CAZ – ceftazidime, COL – colistin, CTX – cefotaxime, FEP – cefepime, S – streptomycin, SH – spectinomycin.  n.d. – not determined  * Transformants *Acinetobacter baumannii* ACI, 1 and 2 were acquired as a result of natural transformation of *A. baumannii* A118 with *Salmonella enterica* Sal25 donor DNA; transformants *Acinetobacter baumannii* ACI-1, ACI-2 and ACI-3 were acquired as a result of natural transformation of *A. baumannii* A118 with *Acinetobacter baumannii* ACI donor DNA. | | | | | | | | | | |
