# Supplementary material for: Large DNA fragment ISEc9-mediated transposition during natural transformation allows interspecies dissemination of antimicrobial resistance genes
Source: Eur J Clin Microbiol Infect Dis. 2025 Mar 28;44(6):1417–24. doi: 10.1007/s10096-025-05113-9 (PMC12116815; doi:10.1007/s10096-025-05113-9)
Supplement: Supplementary file 3 — Supplementary Material 3 [file 10096_2025_5113_MOESM3_ESM.docx]

**Online Resources**

Online Resource 1 – Antimicrobial susceptibility profile of donor, recipient and representative transformant cells.

Online Resource 2 – *Acinetobacter baumannii* A118 transformation frequency (transformants per recipient).
